# Factors associated with uptake of guideline-recommended cardiovascular implantable electronic device management: a nationwide, retrospective cohort study

**DOI:** 10.1017/ash.2023.422

**Published:** 2023-10-25

**Authors:** Sara Young, Hillary J. Mull, Samuel Golenbock, Kelly Stolzmann, Marlena Shin, Rebecca P. Lamkin, Katherine D. Linsenmeyer, Isabella Epshtein, Emily Kalver, Judith M. Strymish, Westyn Branch-Elliman

**Affiliations:** 1 Boston University, Chobanian & Avedisian School of Medicine, Boston, MA, USA; 2 Center for Healthcare Organization and Implementation Research (CHOIR), VA Boston Healthcare System, Boston, MA, USA; 3 Department of Surgery, Boston University Chobanian & Avedisian School of Medicine, Boston, MA, USA; 4 Department of Medicine, VA Boston Healthcare System, Boston, MA, USA; 5 Montclair State University, Montclair, NJ, USA; 6 Harvard Medical School, Boston, MA, USA

## Abstract

Clinical guidelines recommend device removal for cardiovascular implantable electronic device (CIED) infection management. In this retrospective, nationwide cohort, 60.8% of CIED infections received guideline-concordant care. One-year mortality was higher among those without procedural management (25% vs 16%). Factors associated with receipt of device procedures included pocket infections and positive microbiology.

## Background

Cardiovascular implantable electronic device (CIED) infections are rising due to a variety of factors and have a high a high 1-year mortality rate. Management is costly, ranging from an estimated $70,000 to $146,000 per case.^1^ Clinical guidelines recommend using medical (eg, antimicrobial administration) and procedural interventions (eg, device removal) in pocket infections and deeper infections, including cases with lead involvement, and systemic infections/endocarditis.^2–4^

As noted in Sciria *et al*, in the modern era, mortality remains high, in part due to limited uptake of American Heart Association and Heart Rhythm Society clinical management guidelines.^3–5^ Others have called for the urgent need for practice change,^6^ but there are limited data about clinical factors that affect the uptake of guideline-concordant care. Thus, the aim of this retrospective national cohort study was to evaluate the uptake of guideline-recommended procedural management of CIED infections and to identify factors associated with adherence within a retrospective cohort in the Veterans Health Administration (VA) population. A secondary goal was to assess chronic suppressive antimicrobial use as part of CIED infection management.

## Methods

We previously established a database of CIED procedures (eg, pacemaker and cardiac defibrillator device implantations, upgrades, and battery replacements) during the period from 10/1/2016 to 9/30/2019. Trigger-flagged cases underwent manual review by a trained chart reviewer and standardized definitions of CIED infections (pocket and deep infections (eg, systemic/lead involvement/endocarditis)) were applied.^7^ To align with CIED infection management guidelines, only pocket infections and deep infections were included in the final cohort. Pocket infection was defined as infection localized to the generator pocket. Deep infections were defined as an infection of the heart valves, CIED device leads, or evidence of bacteremia/sepsis with clinical signs of CIED involvement.

Demographic details and structured variables were extracted from the VA Corporate Data Warehouse (CDW). Data extracted from the CDW included microbiology orders, collection dates, and results, device procedures performed within 7 days prior to and 365 days after infection (both VA and non-VA care), and 1-year mortality following infection diagnosis. Data about antimicrobial dispenses ≥42 days and ≥90 days following the infection diagnosis were also extracted.

The primary outcome was receipt of an electrophysiology procedure as part of CIED infection management. Data were analyzed using simple descriptive statistics and random effects logistic regression.

All analyses were completed using SAS Enterprise Guide 8.3 software (SAS Institute, Cary NC). This study was approved by the VA Boston Research and Development Committee.

## Results

In total, 309 infections were identified at 68 VA facilities (86.1% pocket and 13.9% deep). The mean age of the cohort was 69.7 years, and 98% were male (Table 1). *Staphylococcus aureus* was the predominant causative organism, and procedures were primarily performed in VA settings (77.7%).

**Table 1.** Patient characteristics and microbiology results among the cohort of VA patients with cardiovascular implantable electronic device infections

	No managing procedure (*n* = 121)	Managing procedure (*n* = 188)
**Age** ***n*** **(%)**		
<65	29 (24.0)	55 (29.3)
65–75	55 (45.5)	93 (48.4)
>75	37 (30.6)	42 (22.3)
**Female** ***n*** **(%)**	2 (1.7)	4 (2.1)
**Race,** ***n*** **(%)**		
Black	21 (17.4)	30 (16.0)
White	96 (79.3)	142 (75.5)
Unknown/Other	4 (3.3)	16 (8.5)
**Hispanic ethnicity,** ***n*** **(%)**	7 (5.8)	12 (6.4)
**Elixhauser index (mean, SD**^**a**^ **)**	14.2 (10.6)	12.9 (11.5)
**Infection type,** ***n*** **(%)**		
Pocket	97 (80.2)	169 (89.9)
Endocarditis	24 (19.8)	19 (10.1)
**Culture type,** ***n*** **(%)**		
No culture	28 (23.1)	19 (10.1)
Blood only	38 (31.4)	39 (20.7)
Wound only	2 (1.7)	10 (5.3)
Both	53 (43.8)	120 (63.8)
**Culture timing,**^b^ ***n*** **(%)**		
No culture	28 (23.1)	19 (10.1)
Before infection date	49 (40.5)	84 (44.7)
On infection date	41 (33.9)	63 (33.5)
After infection date	3 (2.5)	22 (11.7)
**Culture timing,**^c^ ***n*** **(%)**		
No culture	n/a	19 (10.1)
Culture, no managing procedure	n/a	0 (0.0)
Before procedure date	n/a	143 (76.1)
On procedure date	n/a	15 (8.0)
After procedure date	n/a	11 (5.9)
**Microbiology results,** ***n*** **(%)**		
*Staphylococcus aureus*	23 (19.0)	38 (20.2)
Coagulase-negative Staphylococci	10 (8.3)	23 (12.2)
Streptococcal sp.	1 (0.8)	3 (1.6)
Enterococcal sp.	8 (6.6)	7 (3.7)
*Escherichia coli*	3 (2.5)	5 (2.7)
*Klebsiella* sp.	3 (2.5)	8 (4.3)
Pseudomonal sp.	4 (3.3)	8 (4.3)
Other	8 (6.6)	22 (11.7)
Polymicrobial	8 (6.6)	17 (9.0)
Mixed culture, *n* (%)	4 (3.3)	7 (3.7)
Unknown, *n* (%)	71 (58.7)	94 (50.0)
Death within 1 yr of infection, *n* (%)	32 (26.4)	30 (16.0)

a SD = standard deviation.

b Relative to infection diagnosis.

c Relative to managing procedure.

Approximately 20% of patients who developed a CIED infection died within 1 year of the diagnosis (62/309); mortality rate was higher among patients who did not undergo a procedure as part of their CIED infection management (32/121, 24.6% mortality among patients without a device procedure vs 30/188, 16.0% among patients with a procedure).

Among the cohort of infections, 188 (60.8%) received device procedures (169 pocket infections, 63.5% vs 19 deep infections, 44.2%). The majority of procedurally managed infections had both blood and wound/tissue cultures collected (64%). Most cultures were obtained prior to device removal (76% before, 8% same day, 5.6% after, 10% no culture).

**Figure 1.**
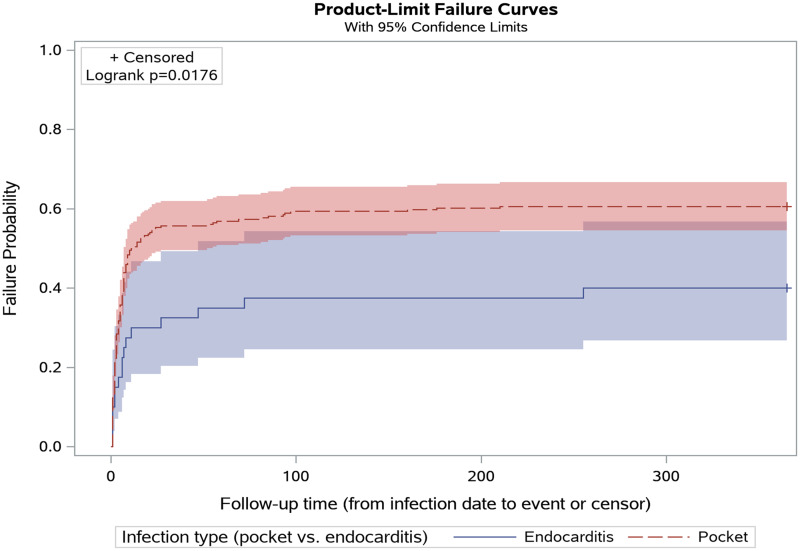
Time in days from infection date to device procedure, stratified by infection type.

Most device procedures (78.2%) were performed within 1 week before or after the infection diagnosis. Among 188 device procedures, the median time to intervention was 2.0 days (IQR 0.0–6.5), with the rate of intervention plateauing abruptly around 90 days postinfection for both infection types (Figure 1). Prolonged antimicrobial use lasting for more than 6 weeks after infection diagnosis was uncommon (68/309, 22%), and no cases of antimicrobial use lasting for greater than 3 months were identified.

In the multivariable analysis adjusted for age and Elixhauser comorbidity index, factors associated with device removal included pocket infection (vs deep infections, adjusted odds ratio (aOR): 2.63, 95% confidence interval (CI): 1.32–5.25) and a positive microbiology result (aOR 1.65, 95% CI: 1.01–2.70).

## Discussion

The AHA and Heart Rhythm Society guidelines recommend complete device removal for all pocket and deep CIED infections with adjunctive antimicrobial therapy.^3,4^ In our large, nationwide cohort study, we found that most patients received guideline-concordant procedural management of their infections. Deep infections and cases without a clear microbiologic diagnosis were associated with purely medical (eg, receipt of antimicrobials only) versus medical and procedural management (eg, receipt of a device procedure in addition to antimicrobials). These findings may be helpful for designing strategies to increase the uptake of guideline-concordant care. Notably, chronic suppressive antimicrobials, commonly used for other infections involving foreign material, were rare.

The 1-year mortality rate was high among all CIED infection cases but higher among those who received medical management only, consistent with prior work. The propensity for pocket infections to be more likely to be managed procedurally may be due to the curative nature of device removal in these localized infections. Patients with deep infections are not cured with device removal alone and may be more clinically unstable and at higher risk for adverse outcomes when undergoing a procedure.

Our findings suggest potential strategies for improving the uptake of guideline-concordant CIED infection management, specifically by improving the collection of bacterial cultures in suspected cases. Improving the appropriate use of diagnostic testing may also have additional positive downstream impacts, such as improving antimicrobial selection and spectrum.

The majority of the CIED infections were caused by *S. aureus*, followed closely by coagulase-negative Staphylococcal species, similar to findings from other studies.^8^ We expected that Staphylococcal species, due to their propensity to adhere to foreign bodies, would be more likely to undergo procedural management than other organisms*. S. aureus* was not associated with higher rates of procedural management of infection; however, statistical power to detect differences between sub-groups was limited.

A strength of our study was our utilization of the VA CDW, which includes granular data about the microbiology of CIED infections and clinical management. Our study has several limitations. The study was retrospective and observational, and residual confounding is always a concern; observed increases in mortality among patients who did not undergo device removal may be secondary to confounding by indication, as patients with higher rates of comorbidities may be less likely to be offered or accept device removal as part of their clinical care. The sample was limited to the VHA population, potentially limiting generalizability. Patients may have received some of their care outside of the VA. This limitation is particularly notable for outpatient intravenous antimicrobials, which are commonly managed by skilled nursing facilities or home care agencies and difficult to capture within the VA EHR. However, we attempted to mitigate this potential missing data by measuring the receipt of oral antimicrobials up to 90 days following the infection diagnosis, which should capture the vast majority of transitions to chronic suppressive antimicrobials.

## Conclusion

Infection is an uncommon but severe complication of CIED procedures, resulting in high morbidity and mortality. Guideline uptake is more common among infections localized to the device pocket than for deeper infections, in which device removal alone is not curative, and in those with positive microbiologic cultures versus those without. These findings may be helpful for identifying ways to improve the uptake of guideline-concordant care and thereby improve clinical outcomes.
